# Effects of a community-based salt reduction program in a regional Australian population

**DOI:** 10.1186/s12889-016-3064-3

**Published:** 2016-05-11

**Authors:** Mary-Anne Land, Jason H. Y. Wu, Adriana Selwyn, Michelle Crino, Mark Woodward, John Chalmers, Jacqui Webster, Caryl Nowson, Paul Jeffery, Wayne Smith, Victoria Flood, Bruce Neal

**Affiliations:** The George Institute for Global Health, PO BOX M201, Missenden Road, Camperdown, NSW 2050 Australia; The University of Sydney, Sydney, Australia; Department of Epidemiology, Johns Hopkins University, Baltimore, USA; The George Institute for Global Health, University of Oxford, Oxford, UK; Royal Prince Alfred Hospital, Sydney, Australia; Deakin University, Melbourne, Australia; New South Wales Health, Sydney, Australia; Faculty of Health Sciences The Sydney University of Sydney, Sydney, Australia; St Vincent’s Hospital, Sydney, Australia

**Keywords:** Salt, Sodium, 24-hour urine, Cardiovascular disease prevention

## Abstract

**Background:**

Salt reduction is a public health priority but there are few studies testing the efficacy of plausible salt reduction programs.

**Methods:**

A multi-faceted, community-based salt reduction program using the Communication for Behavioral Impact framework was implemented in Lithgow, Australia. Single 24-h urine samples were obtained from 419 individuals at baseline (2011) and from 572 at follow-up (2014). Information about knowledge and behaviors relating to salt was also collected.

**Results:**

Survey participants were on average 56 years old and 58 % female. Mean salt intake estimated from 24-h urine samples fell from 8.8 g/day (SD = 3.6 g/day) in 2011 to 8.0 (3.6) g/day in 2014 (−0.80, 95 % confidence interval −1.2 to −0.3;*p* < 0.001). There were significant increases in the proportion of participants that knew the recommended upper limit of salt intake (18 % vs. 29 %; *p* < 0.001), knew the importance of salt reduction (64 % vs. 78 %; *p* < 0.001) and reported changing their behaviors to reduce their salt intake by using spices (5 % vs. 28 %; *p* < 0.001) and avoiding eating out (21 % vs. 34 %; *p* < 0.001). However, the proportions that checked food labels (30 % vs. 25 %; *p* = 0.02) fell, as did the numbers avoiding processed foods (44 % vs. 35 %; *p* = 0.006). Twenty-six percent reported using salt substitute at the end of the intervention period and 90 % had heard about the program. Findings were robust to multivariable adjustment.

**Conclusions:**

Implementation of this multi-faceted community-based program was associated with a ~10 % reduction in salt consumption in an Australian regional town. These findings highlight the potential of well-designed health promotion programs to compliment other population-based strategies to bring about much-needed reductions in salt consumption.

**Clinical trial registration:**

NCT02105727.

**Electronic supplementary material:**

The online version of this article (doi:10.1186/s12889-016-3064-3) contains supplementary material, which is available to authorized users.

## Background

Excess dietary salt causes high blood pressure, which is a major modifiable risk factor for coronary heart disease and stroke [1, 2]. In 2011, the United Nations General Assembly adopted a political declaration that committed Member States to the prevention and control of non-communicable diseases (NCDs) with the objective of reducing premature mortality from NCDs by 25 % by 2025. As part of this effort, a target to reduce salt consumption by 30 % has been set [3] with large health benefits anticipated through effects on blood pressure and vascular diseases [4–6].

In Australia, cardiovascular disease is the leading cause of death and disability and the number one cost to the health sector [7]. Average salt consumption is estimated to be between 7 and 12 g per day (g/day) [8], far exceeding the suggested dietary target of 4 g/day. Like many western populations, it is thought that 75–80 % of the sodium consumed in Australia comes from processed food, with the remainder contributed by home prepared foods or salt added during a meal [9]. Following successful programs in the United Kingdom [10] and Finland [11], current Australian salt reduction efforts are based upon reformulation of the food supply to contain less salt through a public private partnership with the food industry [12]. Intensive nutrition education programs targeting individuals can also reduce dietary salt intake – including teaching individuals to avoid adding salt during food preparation; utilizing salt substitutes (e.g. spices), or choosing lower-salt processed food options [13]. However, individual-based nutrition education are not deemed feasible at the population level because of the resources required [14].

Health promotion programs encourage community-based approaches of lower intensity employing multiple interventions as a strategy for achieving population-level change in behaviors and health [15]. However, whether community-based programs could effectively lower population salt intake remains unknown as only limited data are available to describe efficacy [11, 16–18].

The primary objective of this study was to determine the effect of a multi-faceted community-based salt reduction intervention on mean salt intake, as estimated from measures of 24-h urinary sodium excretion made before and after the intervention was implemented [19]. This paper is responsive to the community-based intervention gap and control recommendations on a global level from the World Hypertension League [20, 21].

## Methods

The study was done in Lithgow, New South Wales, Australia between March 2011 and May 2014. It comprised a baseline survey of the population, an 18 month period of community-based intervention and a follow-up survey of the population done immediately after. Permission to undertake the study was obtained from the Lithgow City Council and the project was approved by the University of Sydney Human Research Ethics Committee. All participants in the survey provided written informed consent. In recognition of the inconvenience associated with the study procedures, participants that completed all components of the survey were provided with an AUD$40 shopping voucher. The study methods have been described elsewhere in detail [19], and we followed the Transparent Reporting of Evaluations with Nonrandomized Designs (TREND) guidelines [22].

### The community-based salt reduction program

The salt reduction intervention targeted the whole community and was based upon the Communication for Behavioral Impact (COMBI) framework [23]. This framework utilizes an integrated communication model to enact community advocacy and impact using five broad components: administrative mobilization and public advocacy to engage key stakeholders; community mobilization; a comprehensive advertising strategy; interpersonal communication; and point of service contact using tools to support interaction (Table 1). The branding of the intervention was “Salt Swap” with key messages including; (1) use *‘FoodSwitch’,* a smartphone application which allows users to scan the barcodes of packaged foods, receive color-coded ratings for four key food components (total fat, saturated fat, sugar and salt) and a list of similar foods that are lower salt healthier choices [24], (2) swap table salt for the salt substitute, which comprises a sea salt blend of 136 mg sodium and 176 mg potassium per serving (0.8 g). This formula results in 70 % less sodium and 22.5 % more potassium than regular salt while retaining good sensory properties [25], (3) use spices and (4) avoid processed foods.

**Table 1:** Salt swap lithgow intervention components

COMBI	Action
Administrative mobilization and public advocacy to engage stakeholders	A series of meetings were held with local government (12 meetings), local doctors (7 meetings) and allied health professionals (6 meetings) all ranging in duration from 0.5–1.0 h.
Community mobilization	A series of meetings were held with five of the largest employers in Lithgow (2 meetings), the local business association (1 meeting), business owners (predominately cafe, pub and restaurant owners–20 meetings) and community groups (11 meetings) all ranging in duration from 0.5–2.0 h.
	Two specific tools (salt substitute and ‘*FoodSwitch’* smartphone application) were used to encourage reduction in salt consumption as well as acting as talking points to engage the community on the topic of the intervention.
Advertising	Local channels of communication including letter box flyers, newspapers, radio and social media were all targeted with information and stories about the program. A series of specific advertising initiatives was scheduled for the period of the intervention during which two letter box drops, seven pieces of print media, one radio interview (replayed), one radio community announcement (replayed) and bi-weekly social media posts were done.
Interpersonal communication	Information booths were established in the two main shopping areas and manned for 80 days for about four hours on each occasion. About 500 individual homes were door-knocked by two team members who each worked on this activity for an average of 25 days. The salt substitute and smartphone application were used as talking points to engage individuals about the importance of reducing salt consumption as well as providing practical ways to achieve a reduction.
Point of service contact using tools to support interactions	The salt substitute was made available for use by consumers free-of-charge at local cafes and restaurants. In addition, government buildings and medical centers held stocks of the salt substitute that consumers could take at no cost. Approximately 8,000 (64 g) packs of salt substitute were provided to the community during the course of the intervention. The smartphone application, ‘*FoodSwitch’* that enable consumers to identify lower salt packaged foods was available as a free download.

Data presented in this table describes the intervention components within the Communication for Behavioural Impact (COMBI) integrated communication model

### Selection of participants for baseline and follow-up surveys

The baseline survey was a random sample of the population aged ≥20 years. Potential participants were drawn from the electoral roll by selecting every 7th person residing within the district. The electoral roll provided the name and address of each potential participant with electronic databases searched to identify corresponding telephone numbers. This sample was supplemented by a volunteer sample recruited from shopping centers, workplaces and community events. For the follow-up survey the participation of the individuals included in the baseline survey was first sought and then supplemented by recruitment of both a further random sample and more volunteers. For the follow-up survey we sampled at random from the telephone directory and also recruited volunteers using the previous methodology. All adults over the age of 20 years residing in the Lithgow area were eligible, with no exclusion based on inter-current illness, use of medications or any other aspect of demography or personal history.

### Data collection

Potential survey participants were mailed invitations to take part in the survey, with an explanation of the purpose of the study, a participant information sheet and a consent form. The exception was volunteers who were provided with an explanation of the purpose of the study in person along with a participant information sheet and a consent form. Thereafter the data collection process was the same for randomly selected individuals and volunteers and for baseline and follow-up surveys.

Within a week of initial contact potential participants were contacted by telephone to determine their willingness to participate and to schedule an interview time. The participants either visited the study office based at the Lithgow community hall or a research assistant made a home visit. Once consent had been obtained data collection was initiated comprising a questionnaire about participant characteristics, a brief physical examination, a questionnaire evaluating consumer knowledge, attitudes and behaviors, and a single 24-h urine collection. The questionnaires and the physical examination were completed at the time of the visit and the 24-h urine collection was scheduled for a time within the following 3–10 days.

The questionnaires were fully structured and administered by a research assistant with all responses based on self-report. The measurement of knowledge, attitudes and behaviors towards salt was based upon a questionnaire adapted from the World Health Organization/Pan American Health Organization protocol [26]. The physical examination comprised measurement of body weight (using calibrated Tanita HD-357 portable electronic scales, USA 0.1 kg) and height (using a calibrated portable stadiometer Wedderburn WS-HRP model, Australia, 0.1 cm) with body mass index (BMI kg/m^2^) then calculated. Blood pressure was measured seated using a manual inflation blood pressure monitor (A&D UA-&704) in triplicate according to the American Heart Association protocol [27]. Physical examination was conducted at any time of the day during work hours (8 am-5 pm) as convenient for the study participant.

A single 24-h urine collection was obtained with the first voided urine upon waking on the day of collection being discarded and participants then collecting all voided urine up to and including the first void the following morning. The times at the beginning and the end of urine collection were recorded. The urine volume was noted and the urinary sodium and potassium concentration in an aliquot was measured by ion-selective electrode with the buffered kinetic Jaffe reaction without deproteinization used for assay of urine creatinine (Cobas Integra 400). Suspected incomplete urine collections (i.e. urinary creatinine < 4.0 mmol/day for women, or < 6.0 mmol/day for men [28] or a 24-h urine collection of < 500 ml for either sex [29]) and suspected over-collections (i.e. urinary creatinine or a urine collection volumes > 3 standard deviations above the population mean [30]) were excluded.

### Outcomes

For each individual, the 24- h sodium excretion value (mmol/day) was calculated as the concentration of sodium in the urine (mmol/L) multiplied by the urinary volume (L/day). The conversion from sodium (mmol/day) to sodium (mg/day) was made by multiplying by 23.0, and the conversion from sodium (g/day) to salt (g/day) was made by multiplying the sodium value by 2.54 [31]. The knowledge, attitude and behaviors questionnaire contained nine questions; four related to knowledge of personal consumption, recommended daily intake and possible harmful effects of salt and five assessing attitudes and behaviors to lowering salt intake. At follow-up, but not at baseline, the questionnaire included questions about knowledge of the program, use of salt substitute and use of the ‘*FoodSwitch’* smartphone application. Depending on the question, the participants answered on a range of different scales such as “rarely, sometimes, often”, “yes, no” and “too much, just the right amount, too little” [32].

### Cost data

The crude cost of the program was recorded manually as either assessment (both baseline and follow-up) or intervention expenses.

### Statistical analyses

The study was designed to provide 80 % power to detect a minimum difference of 0.7 g/day in mean salt excretion in the baseline compared to follow-up surveys, as estimated form 24-h urine samples. This estimate assumed a two-sided *p*-value of 0.05, a standard deviation of urinary salt excretion of 3.6 g/day and required a sample size of 600 participants for each survey assuming non-paired samples.

As pre-specified in the protocol [19], the effects on the primary outcome were estimated by comparing the baseline and follow-up measures of 24-h urinary sodium excretion for the four subsets of survey participants (random sample surveyed at baseline and follow-up, random samples surveyed at baseline or follow-up, volunteers surveyed at baseline and follow-up and volunteers surveyed at baseline or follow-up) and then using a fixed effect inverse-variance weighted meta-analysis to obtain a summary estimate. The analysis was conducted using linear regression for unmatched subjects and generalized estimating equations for matched subjects (i.e. those with 24-h salt data at study baseline and at follow-up). Initial analysis was unadjusted, then two additional models were fitted with the first adjusting for age, sex and BMI, and the second for age, sex, BMI, education, diagnosis of high blood pressure and use of prescribed anti-hypertensive medication. Effects on knowledge, attitudes and behaviors were assessed using prevalence ratios to compare responses between baseline and follow-up. The analytic approach was similar to that used for the primary outcome with results estimated for each subset of survey participants and then pooled using a fixed effects inverse variance weighted meta-analysis to obtain the summary effect. The difference was that a modified Poisson regression with a robust sandwich variance estimator [33] was employed for the unmatched data and a generalized estimating equation for the matched data (to account for the within subject clustering [34]).

Throughout, a *p*-value of 0.05 or less was taken to indicate a finding unlikely to have arisen solely by chance. Statistical analyses were conducted using SPSS for Windows (Version 21, SPSS Inc, Chicago, IL) and STATA for windows (*Release 13.1.* StataCorp. College Station, TX).

## Results

**Fig. 1:**
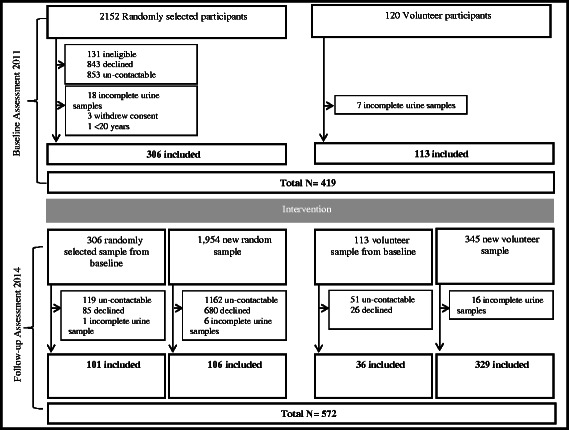
Flow chart. Flow chart shows the research design and recruitment of individuals

There were 2152 randomly selected individuals at baseline of whom 306 provided data (response rate 16 %). At follow-up the corresponding numbers for the random sampling were 106/1954 (response rate 5 %). The baseline sample was supplemented by 120 volunteers and the follow-up sample by 345 volunteers. There were a total of 991 included in the two surveys (513 randomly selected and 478 volunteers) with 137 (101 randomly selected and 36 volunteers) participating at both baseline and follow-up. There were 25 survey participants at baseline and 23 at follow-up for whom the 24-h urine samples were suspected to be incomplete (Fig. 1). Most demographic and clinical characteristics were similar between baseline and follow-up survey participants including age, gender, BMI, smoking status, self-reported health, history of cardiovascular diseases, and lipid and glucose lowering medication use. However, a few differences were noted with the follow-up survey participants being on average more highly educated (*p* = 0.001), more frequently reporting that they had high blood pressure (*p* = 0.02) and more often taking antihypertensive medications (*p* = 0.03) (Table 2).

**Table 2:** Characteristics of participants

	Baseline		Follow-up				
	Random (*n* = 306)	Volunteer (*n* = 113)	Random followed from baseline (*n* = 101)	Random new (*n* = 106)	Volunteer followed from baseline (*n* = 36)	Volunteer new (*n* = 329)	*P* value*
Female (%)	53	61	56	61	61	58	0.25
Age, years range (mean)	20–88 (58)	20–84 (49)	29–90 (64)	21–90 (60)	22–83 (60)	20–89 (52)	0.49‡
Height, cm mean (SD)	168 (10)	167 (8.8)	166 (9.6)	166 (10.2)	168 (8.9)	167 (10)	0.38
Weight, kg mean (SD)	82 (17.1)	84 (20.6)	82 (16.9)	84 (24.1)	86 (26)	82 (19.9)	0.90
BMI, kg/m^2^ mean (SD)	29 (5.1)	30 (6.6)	29 (5.9)	30 (7.0)	30 (8.3)	29 (6.6)	0.45
Systolic bp, mmHg mean	127	124	127	133	131	126	0.10
Diastolic bp, mmHg mean	79	79	78	81	82	80	0.27
Creatinine mmol mean (SD)	12.0 (3.8)	12.0 (4.2)	11.0 (3.6)	12.0 (4.3)	12 (3.8)	12 (4.4)	0.31
Urine volume, ml mean (SD)	1930 (808)	2012 (868)	1957 (859)	2082 (875)	1867 (747)	1930 (823)	0.94
Education (%)							0.01
Secondary	64	56	55	49	50	45	
Tertiary	26	33	40	46	44	47	
Postgraduate	10	12	5	5	6	8	
Health Status (%)							
Very good	50	49	33	30	19	31	0.12
Good	30	24	42	44	42	43	
Fair	20	27	25	26	39	26	
Smoking status (%)							
Current smoker (>1/day)	8	22	7	6	14	18	0.67
Ever smoked (>1/day)	41	53	37	39	50	42	
Alcohol consumption (time since last drink) One week or less	62	43	58	58	40	53	
12 months or more	38	57	42	42	60	47	0.20
Have you ever been told by a nurse of doctor that you have (%)							
High blood pressure	44	30	55	42	50	40	0.02
Heart attack	8	4	11	9	6	3	0.62
Stroke	4	2	6	3	3	2	0.57
Angina	7	4	8	7	11	5	0.93
Diabetes	11	7	13	15	11	11	0.25
Prescription Medication † (%)							
Antihypertensive	22	20	37	31	26	23	0.03
Lipid lowering	16	18	30	22	14	17	0.20
Aspirin	8	3	21	14	9	8	0.01
Glucose lowering	5	9	9	11	12	7	0.13
Any prescription medication	66	53	79	83	60	60	0.10

^*^Age comparison presented is for the new random and volunteer sample with the baseline sample, excluding paired participants (the same random and volunteer sample sampled at baseline at follow-up)

^†^Participants could be taking more than one prescribed medication

^‡^ *p*-value compared mean values across all baseline vs. all follow-up

### Effects on urinary salt excretion

**Fig. 2:**
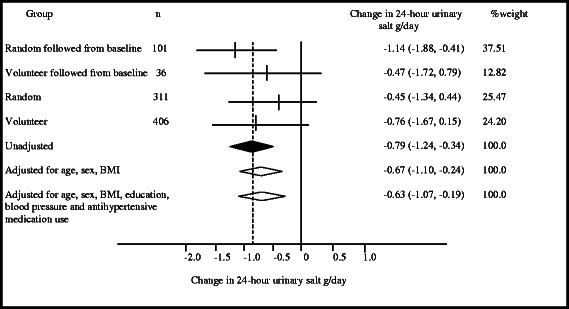
Effect of intervention on urinary salt excretion. Data presented are mean 24-hour urinary salt g/day (95% confidence interval) for continuous characteristics, paired and unpaired t-test for matched and unmatched subjects. Results were then pooled by inverse variance weighted meta-analysis as calculated using a fixed effect inverse-variance weighted method

Mean baseline 24-h urinary salt excretion was 8.8 g/day (standard deviation, SD 3.6 g/day, *n* = 419, Additional file 1: Table S1). At follow-up mean 24-h urinary salt excretion was 8.0 (SD = 3.6 g/day, *n* = 572). The unadjusted difference in mean 24-h urinary salt excretion was −0.8 g/day (95 % confidence interval, −1.24 to −0.34/day; *p* < 0.001) (Fig. 2). There was little evidence that the changes in urinary sodium differed according to sex (*P* for heterogeneity between groups = 0.53, Additional file 2: Figure S1), and there was significant reduction in mean urinary salt in both men (mean, −0.92 g/day, 95 % CI, −1.68, −0.17 g/day, *P* = 0.02) and women (mean, −0.63 g/day, 95 % CI, −1.13, −0.13 g/day, *P* = 0.01). When adjusted for age, sex, and BMI the difference was −0.67 g/day (95%CI, −1.1 to −0.2 g/day; *p* = 0.002) and when also adjusted for education, diagnosis of high blood pressure, and antihypertensive medication use the difference was −0.63 g/day (95%CI, −1.07 to −0.19/day; *p* = 0.005). In all analyses effect estimates were consistent across the four groups of survey participants (all p homogeneity > 0.3). Urine potassium excretion did not differ between follow-up and baseline (*p* = 0.68)), and this finding was consistent for men and women (*P* for heterogeneity between groups = 0.07).

### Effects on knowledge, attitudes and behaviors towards salt

There was no difference in the proportion of subjects between study baseline and follow-up who identified that a high salt diet can cause serious health problems (≥95 % of all subjects at both time points; *p* = 0.76)). Similarly, there was no difference in the proportion of subjects who reported that they consumed too much salt, took regular action to control salt intake, and added salt to food during cooking or at the table (*p* ≥ 0.22 for each).

There was, however, increased knowledge of the recommended upper limit of salt from baseline to follow-up (18 % vs. 29 %; *p* < 0.001), increased understanding of the importance of lowering salt (64 % vs. 71 %; *p* < 0.001) and greater proportions reporting use of spices for flavoring (5 % vs. 28 %; *p* < 0.001) and avoiding eating out (21 % vs. 34 %; *p* < 0.001). At the same time the proportion of participants reporting efforts to control their salt intake by avoiding processed foods fell (44 % vs. 35 %; *p* = 0.006) and marginally lower numbers reported checking food labels (30 % vs. 25 % *p* = 0.02) (Table 3). At follow-up almost all participants (90 %) identified that they were aware of the program to reduce salt intake and about one quarter (26 %) of the participants indicated that they had used the sodium reduced, potassium enriched salt substitute provided. Only three participants reported using the *‘FoodSwitch’* smartphone application.

**Table 3:** Effects of intervention on knowledge, attitudes, and behaviours towards salt

	Baseline	Follow-up	Adjusted prevalence ratio (95 % CI)	
	2011 (*n*=419) Unadjusted prevalence, %	2014 (*n*=572) Unadjusted prevalence, %		*P*-value
Do you add salt to your food at the table?				
Always	21.2	19.4	0.98 (0.75–1.28)	0.88
Do you add salt to food when cooking?				
Always	19.1	20.1	1.14 (0.88–1.48)	0.32
How much salt do you think you consume?				
Too much	27.9	30.1	1.10 (0.89–1.35)	0.37
Maximum salt consumption recommendation?				
Correctly identified as <6 g	18.2	28.7	1.53 (1.19–1.96)	0.001
High salt cause serious health problems?				
Yes	95.0	95.3	1.0 (0.97–1.02)	0.76
How important is lowering salt in your diet?				
Important	63.7	78.2	1.23 (1.13–1.34)	<0.001
Do you do anything to regularly control your salt intake?				
Yes	63.3	60.1	0.94 (0.85–1.04)	0.22
Do you avoid processed foods?				
Yes	44.2	35.3	0.80 (0.68–0.94)	0.006
Do you check food labels?				
Yes	30.0	24.5	0.77 (0.62–0.95)	0.02
Do you buy low salt alternatives?				
Yes	33.9	32.0	0.94 (0.78–1.13)	0.48
Do you use spices?				
Yes	4.8	28.3	5.83 (3.70–9.20)	<0.001
Do you avoid eating out				
Yes	20.8	34.4	1.58 (1.26–1.99)	<0.001

Data shows unadjusted prevalence percentage and adjusted prevalence ratio (95 % confidence intervals) as calculated using a modified Poisson regression for unmatched data and a generalized estimating equation for the matched data

### Costs associated with the intervention

The three key costs associated with the study were human resources (salaries), the shopping voucher ($A40) in recognition of inconvenience, and the pathology expenses. At baseline and similarly at follow-up, due primarily to the increased staff time required for the selection and interaction with the randomly selected individuals the estimated average cost associated with obtaining a valid 24-h urine sample was greater for each participant in the random sample (about $A62 + $A40) compared with each participant in the volunteer sample (about $A31 + A$40) [31]. The intervention was estimated to cost AUD $118,000 including the development and implementation of the COMBI plan, printing and distribution of information pamphlets, manning information stalls and engaging the community through various activities. Some 20,000 samples of the salt substitute were mobilized free of charge and distributed within the community, while this incurred some cost in human and transportation resources, engaging the community to supply the product was a large cost saver. For example, local businesses, government buildings and medical practices all facilitated free samples for community members to try.

## Discussion

This study suggests that a multifaceted, community-based salt reduction program has the potential to deliver a reduction in average population salt consumption. In turn, this would be expected to drive important reductions in morbidity and premature mortality caused by raised blood pressure [35, 36]. The program used an established community intervention framework based upon information dissemination and behavioral modification to achieve results in a short time frame, for a large population with only a moderate investment of resources. If these findings could be shown to be reproducible in other similar communities and sustainable over the long-term, they should be of considerable interest to policy makers seeking to ameliorate the burden of salt-related ill health in similar jurisdictions around Australia and the world [37].

The Communication for Behavioral Impact framework, upon which the intervention program was based, has reported several prior successes in the field of non-communicable diseases [38]. In line with the earlier reports, the program in Lithgow was underpinned by the engagement and participation of the local community that was enabled and coordinated by the project leader. Support and promotion of the project by a large and diverse stakeholder group meant that almost everyone in the study was aware of the salt reduction initiative. Likewise, the reported changes in behavior may be attributed to advocacy undertaken by community organizations, local business, media and health champions who consistently reinforced the key message of the program. The impact of these activities is demonstrated by the findings from the questionnaires.

There were two tools used to support community engagement and it is likely that one, the smartphone application ‘*FoodSwitch’,* had little impact with very few community members reporting usage. There are potentially several reasons for the low uptake of the application including, the low use of smartphones, lack of time to adopt new technology, and difficulty in understanding the application. The evidence supporting a direct role of the salt substitute in the observed reduction in salt intake is also weak given the absence of a rise in urinary potassium excretion. However, there is little doubt amongst those on the ground implementing the program that the salt substitute was an invaluable tool for engaging with individuals. The salt substitute provided a highly tangible talking point for initial discussions with community members [39] and was a constant reminder of the program for large numbers who had the salt substitute in their home and for many others who saw containers in local cafes, restaurants and stores. Its presence in local medical practices and the capacity of medical practitioners to provide free samples to patients likely further reinforced the acceptability of the broader intervention program.

Our findings suggest improvements in several important knowledge, attitudes and behavior parameters in relation to dietary salt following our intervention. Most prominent among these included a nearly 6 fold increase in the number of participants who reported use of spices during cooking (corresponding to one of the key messages of our intervention program) and a significant 15 % increase in subjects who avoided eating out. Emerging data suggest that increased spice use helps to facilitate reduction in salt intake [40]. Similarly, Australians spend on average ~30 % of their household food budget on dining out and fast foods, which contain high levels of salt, so avoiding eating out may contribute to reduction in salt consumption [41, 42]. Conversely, the decreased proportions of participants reporting efforts to control salt intake by avoiding processed foods or checking labels was unanticipated. The latter result for checking of labels may be a chance finding because the *p*-value was not extreme and there were multiple comparisons made. However, the result for processed foods seems unlikely to have arisen by chance and may be the result of the intervention program changing the communities understanding of the breadth of products that constitutes a processed food.

At the same time as this program was implemented in Lithgow there have been ongoing national efforts to reduce population salt intake through reformulation of the food supply. Previous experience in the United Kingdom suggests that setting clear, voluntary reformulation targets for processed foods can achieve significant reductions in population-wide salt intake [10]. The Australian government’s Food and Health Dialogue has followed this model and set sodium reduction targets for nine food categories, three of which were due to have been implemented by the conclusion of the Lithgow intervention period (bread, ready to eat breakfast cereals and processed meats) [12]. However, the small reductions in average salt content achieved for these three food categories by the due date of December 2013 is unlikely to fully explain the differences in salt consumption we observed in Lithgow and it is unlikely that reductions in the other targeted food categories could either [43]. Furthermore, a parallel evaluation of salt consumption in Victoria (Australia) done over the same time period in a community where there was no intervention has identified no difference in salt intake using a similar before and after study design [44]. This negative control provides some reassurance that while there were several possible limitations with the current research it appears somewhat likely that it identified a real effect of the intervention done in Lithgow. Nonetheless, compared to a community-based approach, system wide reformulation of processed food has the potential for population-wide impact, and future studies are needed to model the impact of the Food and Health Dialogue on salt consumption in Australia.

### Strengths and limitations

This study benefits from the assessment of salt intake based upon the gold standard method of 24-h urine collections, allowing precise and reliable determination of changes in average population level salt intake. Another key strength is the simultaneous evaluation of knowledge, attitudes and behavior towards salt consumption which provides insight into other crucial parameters affected by the intervention program. Although residual confounding cannot be excluded, detailed and standardized collection of demographic and medical characteristics allowed careful multivariate adjustment for these factors, which did not substantively change the estimated effect size.

This study also has several limitations. The intervention duration was short and it is not clear whether the observed benefits represent the full potential of this type of program or whether they could be sustained in the longer term. In addition, because the intervention consisted of multiple components that were implemented simultaneously it is hard to quantify the relative contributions of each to the success of the program. We also did not quantify the amount of reduction in salt intake that may have occurred due to reformulation of processed foods as part of the Food and Health Dialogue, although the very limited number of food categories affected by this initiative and the limited progress observed suggest it is likely to only account for a small portion of the reduction in salt intake [43]. Although there was little evidence to suggest systematic differences in key demographic or medical characteristics comparing participants who volunteered to those randomly selected, we acknowledge the mixed sampling method (as a result of a low survey response rate) may limit the overall generalizability of our results to other regional populations in Australia. Nonetheless comparable levels of salt intake and of knowledge and reported behaviors in other Australian surveys [45–50] using a range of different instruments suggest that the baseline findings are likely also to be valid outside of Lithgow. The extent, however to which the intervention findings are generalizable to communities with different socio-demographic characteristics to Lithgow is unknown, and suggest the need to further evaluate COMBI-based interventions in other settings. Finally, the study did not examine effects on blood pressure levels and other clinical outcomes because this would have required a much larger sample size than was possible. The small difference between baseline and follow-up in the proportions reporting high blood pressure and use of prescribed medications could be a consequence of the program increasing community awareness of salt and raised blood pressure [2, 4, 51].

## Conclusion

In conclusion, this study provides a basis for the development and implementation of community-based programs for salt reduction, although the efficacy and sustainability of this program needs to be confirmed in additionally robustly designed studies. Since the Lithgow initiative used the established Communication for Behavioral Impact framework it should be possible for other communities around Australia and the world to develop programs that follow the principles of this framework, and yet allow adaptations tailored to the circumstances of their particular community.

### Consent to publish

Not applicable.

### Availability of data and materials

The dataset(s) supporting the conclusions of this article will be made available upon request.

## Additional files

Additional file 1: Table S1. Baseline and follow-up urinary salt and potassium excretion (g/day). (PDF 7 kb) Additional file 2: Figure S1. Effect of intervention on urinary salt excretion (g/day). (DOCX 50 kb)
