# Supplementary material for: Effects of a community-based salt reduction program in a regional Australian population
Source: BMC Public Health. 2016 May 11;16:388. doi: 10.1186/s12889-016-3064-3 (PMC4864903; doi:10.1186/s12889-016-3064-3)
Supplement: Additional file 1: Table S1. — Baseline and follow-up urinary salt and potassium excretion (g/day). (PDF 7 kb) [file 12889_2016_3064_MOESM1_ESM.pdf]

SUPPLEMENTARY TABLE 1.BASELINE AND FOLLOW-UP URINARY SALT AND POTASSIUM EXCRETION (g/day)

grams per day (g/day)	**Baseline** n=419	**Follow-up** n=572
Salt	8.8 (3.6)	8.0 (3.6)
Potassium	1.86 (0.6)	1.86 (0.7)

Table Data presented are mean 24-hour urinary salt and potassium g/day (standard deviation) at baseline and follow-up.
