# Supplementary figures and images for: Effects of a community-based salt reduction program in a regional Australian population
Source: BMC Public Health. 2016 May 11;16:388. doi: 10.1186/s12889-016-3064-3 (PMC4864903; doi:10.1186/s12889-016-3064-3)

**SUPPLEMENT FIGURE 1. EFFECT OF INTERVENTION ON URINARY SALT EXCRETION (g/day)**


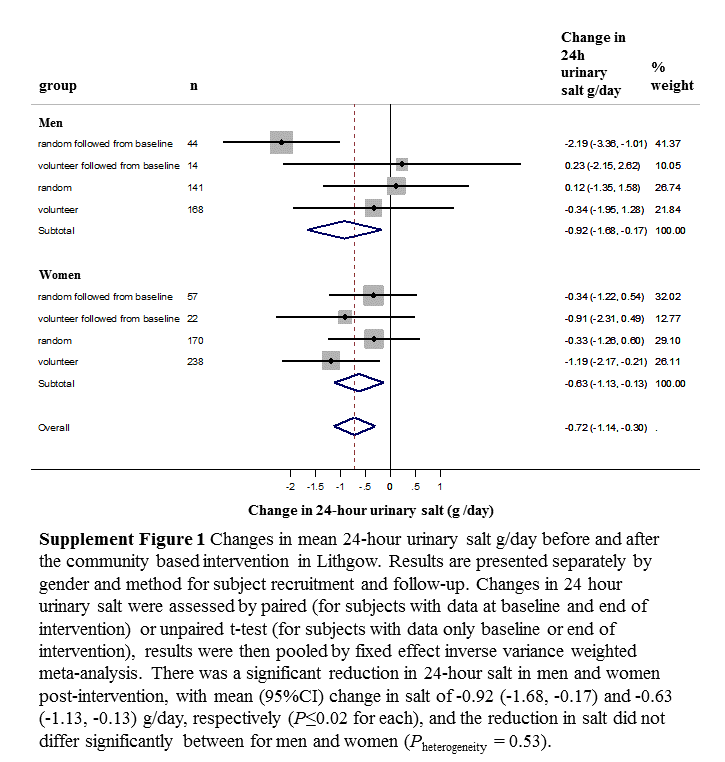

Supplement: Additional file 2: Figure S1. — Effect of intervention on urinary salt excretion (g/day). (DOCX 50 kb) [file 12889_2016_3064_MOESM2_ESM.docx]
